# Therapeutic Bispecific T-Cell Engager Antibody Targeting the Transferrin Receptor

**DOI:** 10.3389/fimmu.2019.01396

**Published:** 2019-06-21

**Authors:** Mingpeng Fu, Qi He, Zilong Guo, Xiaoran Zhou, Heli Li, Liang Zhao, Hongling Tang, Xiaoqi Zhou, Huifen Zhu, Guanxin Shen, Yong He, Ping Lei

**Affiliations:** ^1^Department of Immunology, School of Basic Medicine, Tongji Medical College, Huazhong University of Science and Technology, Wuhan, China; ^2^Department of Transfusion Medicine, Shandong Provincial Hospital Affiliated to Shandong University, Jinan, China; ^3^Department of Nuclear Medicine and PET Center, Zhongnan Hospital of Wuhan University, Wuhan, China

**Keywords:** bispecific antibodies, T-cell engager, transferrin receptor, tumor immunotherapy, solid tumor

## Abstract

Bispecific T-cell engager antibodies (BiTE) have been explored as a means to recruit cytolytic T cells to kill tumor cells. The transferrin receptor (TfR) is highly expressed on the surface of rapidly proliferating tumor cells. Therefore, it holds great potential in T cell redirecting therapies. In this research, we developed a BiTE targeting TfR and CD3 (TfR-BiTE) and studied its therapeutic impact on TfR-positive cancer. TfR-BiTE had the ability to induce the selective lysis of various TfR-positive cancer cells through the activation of T cells, the release of cytokines, and then the coming proliferation of T cells, whereas TfR-negative cells were not affected. In a subcutaneous HepG2 xenograft model, low concentrations of TfR-BiTE inhibited tumor growth. Overall, these results reveal that TfR-BiTE can selectively deplete TfR-positive HepG2 cells; hence, it represents a novel immunotherapeutic approach for the treatment of hepatocellular carcinoma.

## Introduction

Redirecting the activity of T cells by bispecific antibodies against tumor cells, independent of their intrinsic antigen-specific T cell receptor (TCR) recognition, is a potent approach to treat cancer (1–4). The concept of bispecific T-cell engager (BiTE) is based on recognition of an antigen on tumor cell and simultaneous binding to the CD3 epsilon chain (CD3ε) within the TCR complex on T cells (5, 6). It bridges malignant tumor cells directly to CD3-positive T cells, bypassing TCR specificity, and major histocompatibility complex (MHC) class I molecules (7–9). This triggers T cell activation, including the release of cytotoxic molecules, cytokines, and induction of T-cell proliferation (10). In other words, BiTE antibodies direct the host's immune system and activate specifically cytotoxic T cells to kill cancer cells. Blinatumomab (Blincyto), which is reactive with the pan B cell antigen CD19, has been approved by the FDA for treatment of B cell neoplasms in 2014 (11–13).

Therapeutic BiTE antibodies are developed to direct to high-density, cell surface proteins (14). Transferrin receptor (TfR), a disulfide-linked transmembrane glycoprotein homodimer, is an essential protein involved in iron uptake and the regulation of cell growth (15). It is expressed with high levels on rapidly proliferating tumor cells, as well as circulating tumor cells, tumor precursor cells, or cells that have been activated during tumorigenesis (16–18). In addition, the TfR expression level is associated with tumor typing and poor prognosis (16, 19). This makes TfR an attractive target for cancer immunotherapy. TfR Abs have been explored for the treatment of various tumors (20–22). Our previous studies also demonstrated that TfR Abs could recognize tumor cells with high efficiency *in vitro* and ^131^I-TfR Ab displayed a feature of specific accumulation at tumor tissue *in vivo* (23–25). Also these TfR Ab-modified therapeutic agents exhibit tumor-specific cytotoxic activities (26–29).

Here, we describe a TfR bispecific T-cell engager (TfR-BiTE), an anti-human TfR and anti-human CD3 recombinant antibody, a tandem scFv, as T cell–recruiting therapeutics for TfR^+^ malignancies. We provide evidence of potent *in vitro* and *in vivo* killing activity for TfR positive HepG2 cells. This study highlights a new approach in tumor immunotherapy and provides the rationale for treatment of TfR-positive tumors.

## Materials and Methods

### Cell Culture

HepG2, Luc-HepG2, HT1080, and HepG2.215 cells were stored in our lab. MX-1 cells were kindly provided by Professor Xiyun Yan (Chinese Academy of Sciences, Beijing, China). HepG2, HepG2.215, and MX-1 cells were cultured in DMEM. HT1080 and peripheral blood mononuclear cells (PBMCs) were cultured in RPMI 1640 supplemented with 10% fetal bovine serum (FBS), penicillin, and streptomycin, at 37°C in an atmosphere of 5% CO_2_. PBMCs were isolated from healthy donors by Ficoll density centrifugation. Stably transfected CHO-DG44 cells were cultured in CD OptiCHO^TM^ Medium (#12681-011, Gibco, USA) supplemented with L-glutamine (40 mL/L, #25030-081, Gibco, USA) and 1 μM MTX (Sigma-Aldrich, Saint Louis, MO, USA). CD3^+^ cells were depleted from PBMCs using CD3 MicroBeads (human, #130-050-101, Miltenyi Biotec, Germany) according to the manufacturer's recommendation.

### Construction of TfR-BiTE

The eukaryotic plasmid pOptiVEC-TfR-CD3-His encoding the full length of TfR-BiTE and was constructed as follows. The *NheI*-signal-CD3-scFv-*BamHI* fragments were amplified from plasmid pET-28(a)-CD3-scFv (preserved by our lab) by PCR with primers pairs (p1: CTAGCTAGCACCGGTTCCCAGGTCCAGCTGC; p2: CGCGGATCCTTTTATTTCCAACTTTG). Then, the *NheI*/*BamHI*-cleaved PCR fragments were subcloned into plasmid pOptiVEC-TfR-scFv-His (preserved by our lab) to obtain pOptiVEC-TfR-CD3-His (Supplementary Figure 1).

### TfR-BiTE Production

CHO-DG44 cells were transfected with the TfR-BiTE expression vector, and stable expression was achieved by standard drug selection with MTX. TfR-BiTE was purified from the culture supernatant using a HisTrap excel column (#17-3712-05, GE Healthcare, Germany) attached to a peristaltic pump. In brief, DG44 cell-conditioned culture media was clarified and loaded onto the column. The column containing bound TfR-BiTE was washed with 15 mM imidazole and then eluted with 500 mM imidazole. An Amicon® Ultra-15 30 K centrifugal filter device was used to concentrate and dialyze the protein against PBS for three times.

TfR mAb (preserved by our lab), CD3 mAb (preserved by our lab), and mAb Mix (a mixture of 500 ng/ml TfR mAb and 500 ng/ml CD3 mAb) were set as negative controls.

### Binding of TfR-BiTE to Target Cells

The binding ability of TfR-BiTE to TfR-expressing cells (HepG2) and CD3-expressing T-cell (in PBMCs) was assessed. 1 × 10^5^ HepG2 cells and 1 × 10^6^ PBMCs were incubated with TfR-BiTE (10 nM) or equimolar amounts of TfR mAb and CD3 mAb at 4°C for 1 h. Then cells were washed with 4 ml cold PBS and incubated with the anti-6X His tag antibody[HIS.H8] (1:200, #ab18184, Abcam, Cambridge, UK) at 4°C for 1 h. Cells were rewashed and incubated with the PE Goat anti-mouse Ig (1:200, #550589, BD Bioscience, USA). After washing twice in cold PBS, the cells were resuspended in 250–300 μl of PBS and analyzed by Flow Cytometry Analyzers (FCM, LSR II or Verse system, BD Biosciences, Franklin Lakes, NJ, USA). The results were analyzed using FlowJo (LLC, CA, USA) software.

### Confocal Microscopy

The ability of TfR-BiTE to crosslink HepG2 and T cells was assessed as follows. 3 × 10^4^ HepG2 cells adherent to round coverslips (sterilized) were incubated with suspension PBMCs in the presence of TfR-BiTE or control antibodies indicated for 80 min in a 24-well flat-bottom plate. After thrice washes, the cell mixture was fixed and stained with Hoechst 33342 (diluted 1:1000 in PBS). Then the round coverslips were mounted onto glass slides with antifade reagent and examined using a confocal laser scanning microscope (FV1000; Olympus, Tokyo, Japan).

### Cytotoxicity Assay

Target cells (HepG2, HT1080) and CFSE-labeled PBMCs were co-cultured (E:T = 10:1) with or without TfR-BiTE for 24 or 48 h. After that, the cells were stained with 7-AAD (BioLegend, SanDiego, CA, USA) and then analyzed by FCM within 1 h. The CFSE^−^/7-AAD^+^ cells were counted as lysed target cells, while the CFSE^−^/7-AAD^−^ cells as viable target cells. The percentage of specific lysis was calculated as follows: specific lysis% = (percentage of viable cells in the absence of TfR-BiTE – percentage of viable cells in the presence of TfR-BiTE)/(percentage of viable cells in the absence of TfR-BiTE)i × 100.

### CFSE Proliferation Assay

1 × 10^5^ tumor cells were incubated with CFSE-labeled PBMCs (E:T = 10:1) in medium supplemented with TfR-BiTE at the concentrations indicated for 48 h. Thereafter, the cell mixture was cultured in the medium without TfR-BiTE for an additional 72 h. Then cells were stained with a mixture of V450 mouse anti-human CD3, APC-Cy7 mouse anti-human CD4, and V500 mouse anti-human CD8 (BD Biosciences). After washing, the cells were stained with 7-AAD for 15 min. Proliferation on 7-ADD^−^/CD3^+^/CD4^+^ and 7-ADD^−^/CD3^+^/CD8^+^ T-cell subsets was determined by flow cytometry.

### *In vivo* Efficacy Studies

All *in vivo* experimental procedures were approved by the Ethics Committee of Tongji Medical College of Huazhong University of Science and Technology. Severely immunocompromised NCG mice (female, 3–4 weeks, purchased from the Nanjing Biomedical Research Institute of Nanjing University) were subcutaneously inoculated with 1 × 10^6^ Luc-HepG2 cells. On day 7, 1 × 10^7^ PBMCs were infused via tail injection. Six hours later, 20 μg TfR-BiTE or control mAb mixture was injected intravenously. Over the treatment course, PBMCs were given once, and BiTE was given every day for 7 days. The tumor volume and the mouse weight were measured every second day. When the tumor volume was ~2,000 mm^3^, the mice were euthanized, and the tumors were harvested and photographed. Tumor infiltrated T-cells were analyzed by immunohistochemistry using anti-human CD3 (Kit-0003, Maxim biotechnologies, China). Hepatotoxicity and nephrotoxicity induced by TfR-BiTE were evaluated by analyzing liver and kidney cross-sections stained with haematoxylin and eosin.

### Statistical Analyses

Data were analyzed using the unpaired two-tailed Student's *t*-test. All values in the study were expressed as means ± SD. Statistics were computed with PRISM6 software (GraphPad Software Inc.). Differences of *p* < 0.05 were considered statistically significant.

## Results

### Identification of Recombinant Bispecific Antibody

**Figure 1:**
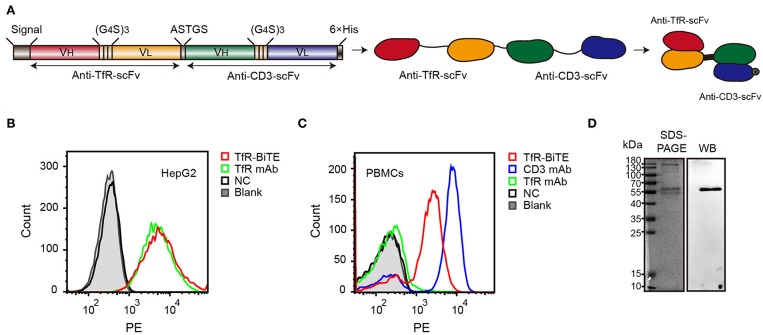
Identification of TfR-BiTE. **(A)** Schematic representation of the TfR-BiTE constructs. Each scFv was composed of immunoglobulin variable heavy chain (V_H_) and immunoglobulin variable light chain (V_L_) domains, which were linked by a 15-residue peptide linker (G_4_S)_3_ (light brown boxes). The silver box represents the short linker peptide (ASTGS), whereas the gray and black boxes represent the signal peptide and the 6×His tag, respectively. **(B,C)** Binding of TfR-BiTE with HepG2 cells (TfR^+^) and unstimulated PBMCs (TfR^−^ and CD3^+^) were detected using anti-His tag mAb by flow cytometry analysis. PBS was set as the negative control (NC). **(D)** SDS-PAGE and Western blot analysis of TfR-BiTE. The migration distances of the molecular mass markers are indicated in kilodaltons (kDa).

The alignment of TfR-BiTE is shown in Figure 1A. TfR-BiTE was constructed by linking single-chain variable fragments (scFv) of anti-TfR mAb and anti-CD3 mAb in tandem. Heavy and light chain variable fragments from both mAbs were linked with (glycine 4-serine) 3 linkers. For the convenience of gene clone, the two scFvs were linked by a 5-residue peptide linker (ASTGS) to encourage flexibility between 2 scFv regions. A C-terminal His × 6 Tag was included for metal affinity chromatography. The TfR-BiTE was constructed into the pOptiVEC vector encoding dihydrofolate reductase (DHFR). The vector was stably transfected into CHO-DG44 cells, which lack DHFR expression (DHFR^−/−^).

Data demonstrated that TfR-BiTE bound to CD3-expressing T cells and TfR-expressing HepG2 cells. Moreover, this binding was comparable with TfR mAb but inferior to CD3 mAb on a molar basis (Figures 1B,C). SDS-PAGE showed fusion proteins were successfully expressed and western blot displayed a specific protein band with an approximate molecular weight of 56 kDa in the purified antibody which was consistent with the predicted molecular weight of BiTE (55 kDa) (30) (Figure 1D). In addition, we have analyzed the bands with Image J software and determine that the purity of BiTE is around 45% in purified products. Mass spectrometry (LC-MS/MS) analysis showed that the unique peptide sequence “ASGYTFTNYYMHWVR” of BiTE could be detected in the purified protein (Supplementary Figure 2).

### TfR-BiTE Is an Engager Between Targets and T Cells

**Figure 2:**
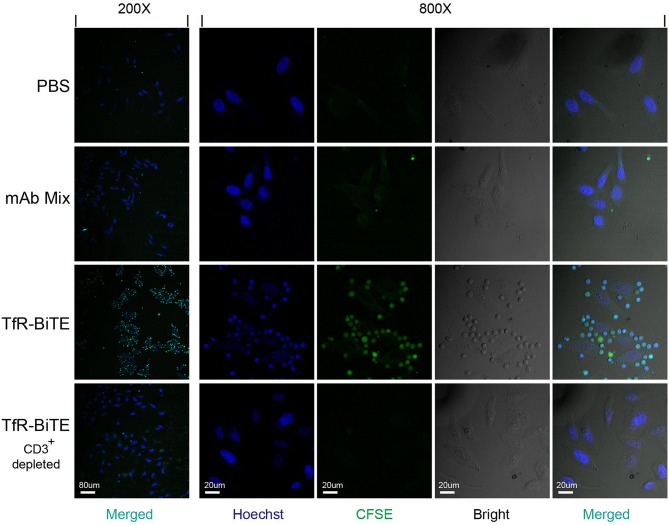
TfR-BiTE is an engager between targets and effector cells. 3 × 10^4^ HepG2 cells adherent to coverslips were incubated with CFSE-labeled PBMCs depleted with or without CD3^+^ cells in the presence of TfR-BiTE or mAb Mix for 80 min. After thorough washes, the cells on coverslips were fixed and stained with Hoechst. Rosette-like configurations were examined using a confocal microscope.

In order to detect whether TfR-BiTE could simultaneously bind to TfR-expressing cells and CD3-expressing cells, adherent HepG2 cells (TfR^+^) were co-cultured with suspension CFSE-labeled PBMCs in the presence of TfR-BiTE or TfR/CD3 mAb mixture. If T cells in PBMCs could be engaged with HepG2 cells by TfR-BiTE, they would also adhere to the culture surface and would not be removed by thorough washes. Images showed that in TfR-BiTE treated groups, many CFSE-labeled lymphocytes were still present and were arranged around the central HepG2 cell to form a cluster that looked like a flower. However, in PBS and mAb mixture treated groups, CFSE- labeled lymphocytes were washed away and absent on the culture surface. No rosette-like configurations were formed. For reconfirmation of the binding specificity of TfR-BiTE with CD3 molecules, HepG2 cells were co-cultured with CD3^+^-depleted PBMCs in the presence of TfR-BiTE. As expected, no green fluorescence-emitting cells and rosette-like configurations could be observed through a microscope (Figure 2). These data suggested that T cells were required for the activity of TfR-BiTE, which could act as an engager between TfR^+^ targets and T cells.

### TfR-BiTE Mediates Cytotoxicity to TfR-positive Tumor Cells *in vitro*

**Figure 3:**
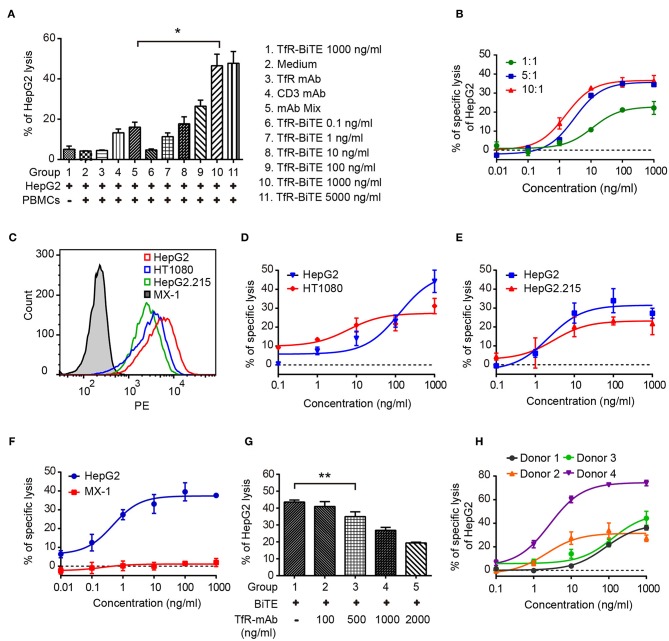
TfR-BiTE mediates cytotoxicity to TfR-positive tumor cells *in vitro*. **(A)** CFSE-labeled PBMCs and HepG2 cells (E:T = 10:1) were incubated with antibodies indicated for 24 h. Then cells were stained with 7-AAD. CFSE^−^ 7-AAD^−^ cells were calculated as viable tumor cells. The percentage of lysed HepG2 cells was calculated using the formula described in materials and methods. **(B)** PBMCs and HepG2 cells were co-cultured with different E:T ratios in the presence of TfR-BiTE at indicated concentrations for 48 h. **(C)** The histogram showed the expression of TfR in four tumor cell lines measured by FCM. **(D–F)** TfR-BiTE (1,000 ng/ml) potency against four different tumor cells (HepG2, HT1080 HepG2.215, and MX-1 cells, E:T = 10:1). **(G)** TfR mAb competitive binding assay. HepG2 cells and PBMCs were incubated with TfR-BiTE (100 ng/ml) and TfR mAb at indicated concentrations for 48 h. **(H)** TfR-BiTE potency against HepG2 cells incubated with PBMCs from four healthy donors. The percentage of cell lysis was quantitated by FCM and calculated as described in Materials and Methods. Error bars represent SD of triplicates. *p < 0.05; **p < 0.01.

Engagement of TfR-positive tumor cells with T cells would establish immunologic synapses between both cells, resulting in the activation of the CD3 downstream signaling pathway and tumor cells lysis (31, 32). Hence, in the following experiments, cytotoxicity mediated by TfR-BiTE was evaluated. Firstly, *in vitro* potency was assessed against the TfR-positive cell line HepG2. Data showed that HepG2 cells would be lysed in the presence of TfR-BiTE, but not of TfR mAb, indicating the T cell engager role of TfR-BiTE (Figure 3A). And HepG2 cells were lysed in TfR-BiTE dose-dependent and PMBCs number dependent manners (Figure 3B). However, the CD3 mAb and mAb mixture were found to be active in HepG2 cell lysis but not to be equipotent to TfR-BiTE on the same concentration. The explanation was that T cells were temporarily activated and then mediated temporarily lysis to targets following treatment with the anti-CD3 antibody (33).

To explore the impact of TfR expression on TfR-BiTE activity, we tested the potency of TfR-BiTE on human breast cancer cell line MX-1 which expresses TfR at very low levels, and multiple tumor cell lines (HT1080, HepG2.215, HepG2) with various levels of surface TfR expression (Figure 3C). Results manifested that TfR-BiTE was also highly active in the killing of multiple tumor cell lines by PBMCs (Figures 3D,E). Moreover, its killing activities to HT1080 and HepG2.215 were comparable to HepG2, which expresses TfR at higher levels. However, TfR-BiTE had no activity against MX-1 cells that are devoid of TfR expression (Figure 3F). When excessive TfR mAb was supplemented to compete with TfR-BiTE binding with targets, it was observed that TfR-BiTE potency was decreased in a mAb dose-dependent manner (Figure 3G). Together, these data suggest that TfR-BiTE activity is TfR-specific, and the potency of TfR-BiTE depends on TfR expression but does not require a high receptor occupancy on targets.

Figure 3H shows the dose-response curves from four healthy donors. Of these donors tested, 25–75% of HepG2 cells were killed with TfR-BiTE (1,000 ng/ml) within 24 h.

### TfR-BiTE Induces T-Cell Activation

**Figure 4:**
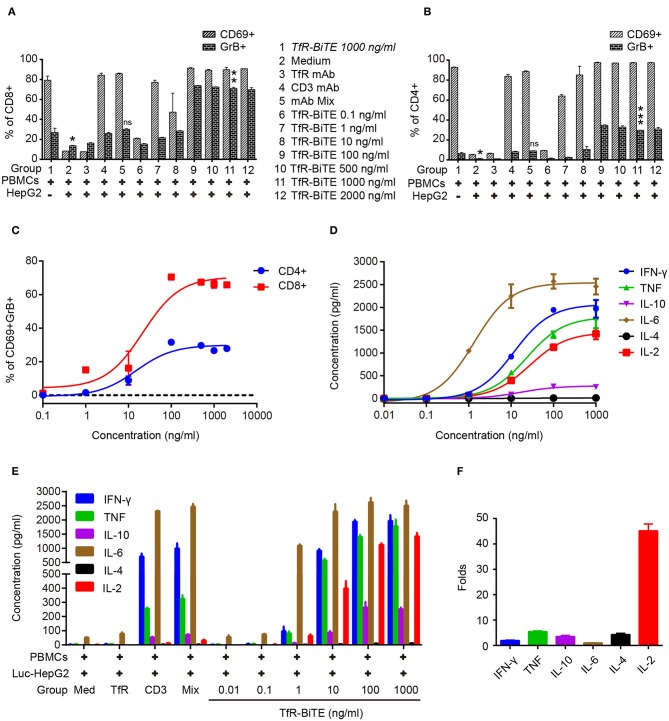
TfR-BiTE induces T-cell activation. HepG2 cells and PBMCs were incubated with various concentrations of TfR-BiTE for 24 h. E:T ratio was 10:1. T-cell activation markers as CD69^+^, GrB^+^ were, respectively, measured and calculated in CD8^+^ **(A)** and CD4^+^ **(B)** subsets. The significance shown in the figure is compared with group 1 (TfR-BiTE 1,000 ng/ml, Italic). CD69^+^GrB^+^ double positive T subsets were analyzed in **(C)**. Cytokine profile released by PBMCs treated with TfR-BiTE **(D)** or control antibodies **(E)** for 48 h. **(F)** Folds of cytokine released (1,000 ng/ml TfR-BiTE group vs. mAb Mix group). Assays were done either in triplicate with average, and SD value plotted or as single-dose response curve representative of multiple assays of different donors. ns, not significant; *p < 0.05; **p < 0.01; ***p < 0.001.

Next, T-cell activation was assessed by the expression of activation markers. Following TfR-BiTE-mediated tumor lysis, both CD8 and CD4 T-cell subsets maintained an activated phenotype as shown by expression of the early activation marker CD69 and cytotoxic granule granzyme B (GrB). By contrast, the CD3 mAb only up-regulated the expression of CD69, but not GrB (Figures 4A,B). We also observed that intracellular GrB up-regulation was more prominent in activated CD8^+^ T-cells than in CD4^+^ T-cells (Figure 4C). As a further hallmark of T-cell activation upon tumor lysis, a number of cytokines were released into culture supernatants, including IFN-γ, TNF, IL-6, and IL-2 (Figures 4D,E). Interestingly, TfR-BiTE induced significantly more IL-2 production (44.18-folds) than control antibodies (Figure 4F). These results demonstrated that T cells were activated by crosslinking with tumor cells through TfR-BiTE.

### TfR-BiTE Promotes T-Cell Proliferation

**Figure 5:**
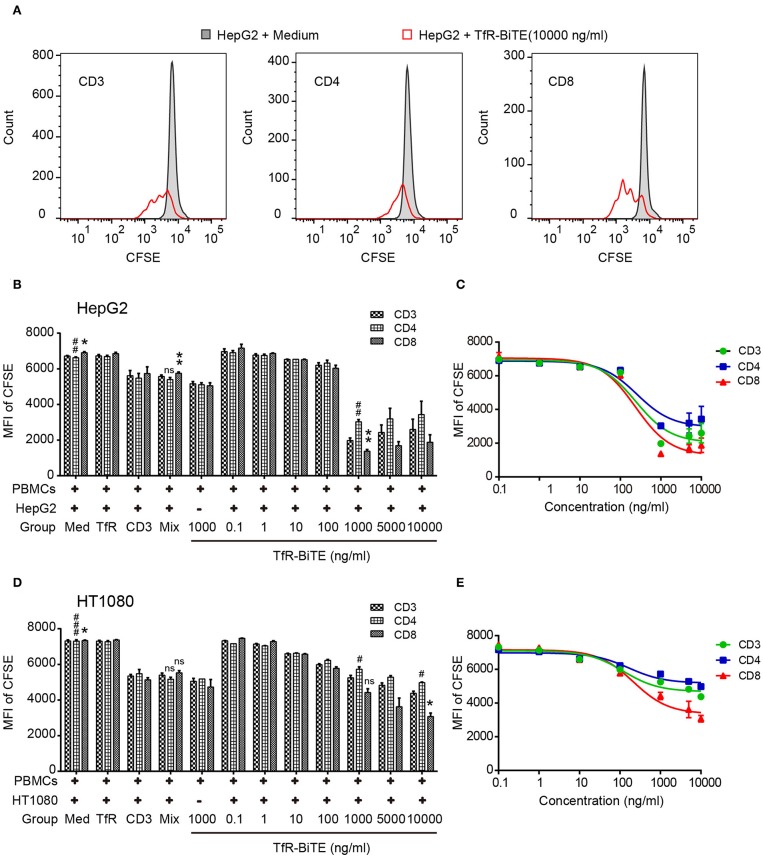
TfR-BiTE promotes T-cell proliferation. HepG2 cells and CFSE-labeled PBMCs (E:T = 10:1) were incubated with indicated antibodies for 5 days. **(A)** Representative histograms of CFSE-labeled T-cell subsets measured by FCM. **(B–E)** Histogram and curve of CFSE-labeled T-cell subsets. The targets were HepG2 cells **(B,C)** or HT1080 cells **(D,E)**. The error bars indicate SD based on triplicates. The significance shown in the figure is compared with the TfR-BiTE 1,000 ng/ml group without target cells. ns, not significant; * or ^#^*p* < 0.05; ** or ^##^*p* < 0.01; ^###^*p* < 0.001.

The use of anti-CD3, in combination with IL-2, leads to a very rapid expansion of activated cell numbers using human PBMCs (34). Given the prominent production of IL-2 induced by TfR-BiTE and the anti-CD3 moiety in the molecule, the proliferative capacity of TfR-BiTE-treated PBMCs was assessed using CFSE proliferation assay. Figure 5 manifested that following TfR-BiTE mediated tumor lysis, the MFI of CFSE in T cells, especially in CD8^+^ T cells, was significantly reduced. When the concentration of TfR-BiTE was increased from 0.1 to 1,000 ng/ml, the MFI of CFSE decreased gradually. However, stimulation with control antibodies resulted in no reduction of CFSE, indicating the proliferative capacity and effective stimulation of TfR-BiTE-treated PBMCs.

### TfR-BiTE Is Potent in Killing TfR^+^ HepG2 Cells *in vivo*

**Figure 6:**
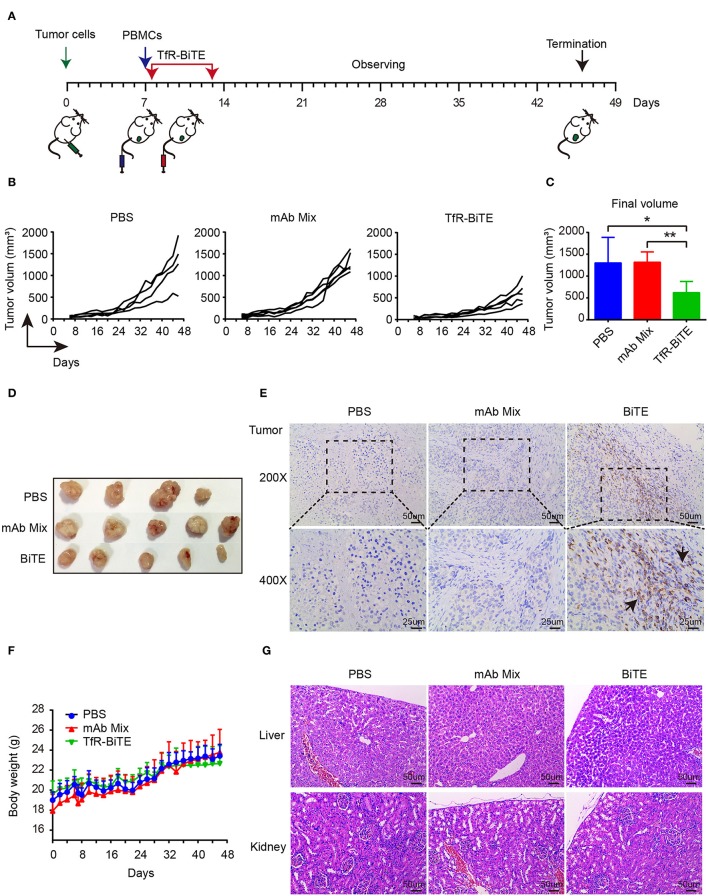
*In vivo* activity of TfR-BiTE in NCG mice. NCG mice were subcutaneously injected with 1 × 10^6^ HepG2 cells on day 0. At day 7, mice were i.v. injected with 10 × 10^6^ PBMCs from healthy human donors. Antibodies (20 μg/mouse) or PBS were administered intravenously 6 h after the transfer of PBMCs. Antibodies were given every day for 7 consecutive days. Tumor volume was measured by a digital caliper. **(A)** Schematic map for the development of xenograft mouse model. **(B)** Tumor growth curve of individual mice (*n* = 4 or 5). Each represents a single mouse. **(C)** The mean tumor volume at the end of the experiment (day 46). **(D)** Images of the tumors harvested from the mice at the end of the experiment. **(E)** Human CD3^+^ cell infiltration in tumor tissues. **(F)** The body weight curves of mice for each group. **(G)** TfR-BiTE elicited no toxicities to liver and kidney. Tissues were collected and stained with haematoxylin and eosin. Scale bar indicates 50 μm. Abbreviations: mAb Mix, TfR mAb, and CD3 mAb mixture. *p < 0.05; **p < 0.01.

The *in vivo* antitumor activity of TfR-BiTE was assessed in immunodeficient NCG mice (Figure 6A). Data manifested that the tumor growth curves of PBS and mAb Mix groups were steeper than that of TfR-BiTE group (Figure 6B), indicating the high effectiveness of TfR-BiTE in preventing tumor growth (Figures 6C,D). Of note, it was observed that the tumor volume among the three groups did not show significant differences at the early stage of therapy. Approximately at day 32 the tumor volume in TfR-BiTE treated mice began to be significantly smaller than those in the other two groups (Figure 6B). The process of T cells trafficking to and accumulating in solid tumor sites is complicated and crucial for redirected T cell-mediated immunotherapy (35). To further characterize the mode of TfR-BiTE against TfR^+^ targets, T lymphocytes that infiltrated into tumor sites were investigated. Immunohistochemistry images showed that there was a clear infiltration of human CD3^+^ lymphocytes in TfR-BiTE treated tumor tissues but not in the control groups (Figure 6E), suggesting that infused T lymphocytes were effectively recruited to the solid tumor sites and were activated by the engagement with targets via TfR-BiTE and then performed their cytotoxic activities.

Finally, the biological safety of this therapy was evaluated. Data showed that, following TfR-BiTE and PBMCs infusion, the mice did not develop a progressively worsening disease characterized by weight loss (Figure 6F). Given the liver and kidney are the main metabolic organs for BiTE antibodies (36), the liver and kidney damages potentiated by biotechnologically produced TfR-BiTE were concerned. Images revealed that no obvious lesions were observed in the liver and kidney cross-sections (Figure 6G), suggesting no hepatotoxicity and nephrotoxicity elicited by this biotechnology-derived product and this kind of therapy.

## Discussion

Among immunotherapies involving CTLs, BiTE antibodies may be a more promising approach. Therapeutic BiTE antibodies have been developed to date are directed to well-known, high-density, cell surface proteins (37). The high and wide expression of TfR on proliferating tumor cells (18) makes it an attractive target for cancer immunotherapy. In the present study, we described the generation of a BiTE to redirect T cells to TfR-positive malignancies (TfR-BiTE). We demonstrated the feasibility, and potential application of the TfR-BiTE construct derived from a TfR mAb and a CD3 mAb, a novel bispecific antibody for the targeting of TfR-expressing tumors with a classic structure such as Blinatumomab (anti-CD19/CD3 bispecific T-cell engager).

The structure of an antibody is related to its biological properties. The extensively studied macromolecular IgG-like bispecific antibodies lead to increased antibody retention *in vivo* (38). However, BiTE without Fc fragments was preferred because of its small molecular weight and the resulting sufficient tumor penetration in solid tumors (39). Although Compte et al. (40) demonstrated that two-chain structured BiTE was better than single-chain structured one, our study suggested that single-chain tandem TfR-BiTE was better in binding with the target cell (Supplementary Figure 3). The binding specificity of both moieties were confirmed as the TfR-BiTE potency was abrogated to TfR-negative tumor cells, was blocked by TfR mAb and no rosette-like configurations was observed in CD3^+^ depleted PBMCs. These results verified that TfR-BiTE activity is TfR and CD3 double specific. The potency of TfR-BiTE requires T cells and depends on TfR expression.

The mechanism of BiTE action attributes to a direct interaction between T cells and the mAb-specific cellular target that triggers T-cell activation and cytotoxicity (41, 42). In our studies, the close proximity of T cells and tumor cells engaged by TfR-BiTE was verified by the formation of rosette-like structures, which was consistent with the TfR-BiTE-mediated tumor cell killing. The potency of TfR-BiTE to activate T cells was confirmed by the up-regulation of activation markers (CD69 and granzyme B), cytokine secretion (IFN-γ, TNF, IL-2, and IL-6), as well as the proliferation of both CD8 and CD4 T-cell subsets. Of note, the eminent production of IL-2 potentiated by TfR-BiTE can facilitate the expansion of the number and function of antigen-selected T cell clones, thus plays a key role in enduring cell-mediated immunity (43).

The *in vivo* anti-tumor effect of the antibody was influenced by the size of the tumor at the time of treatment, the timing of administration, the number, and frequency of the T cells injection. In general, cancer cells are combined with immune effectors to be inoculated into immunodeficient mice, followed by the administration of a test agent before the formation of the tumor (31). In our studies, PBMCs were inoculated and BiTE antibodies started to be administered when tumor volume reached approximate 50 mm^3^. The increased number of infiltrating lymphocytes and better tumor suppression were still observed, suggesting that TfR-BiTE can effectively inhibit tumor progression. In such cases, tumor inhibition was accompanied by an increased number of tumor-infiltrating leukocytes (CD3^+^ cells), reflecting that T cells were effectively recruited from the peripheral blood into the tumor bed to inhibit the growth of TfR-positive xenografts.

However, as TfRs are also present on endothelial cells of the brain although not on endothelial cells elsewhere in the body (44), different TfR bispecific antibodies have been developed to act as a transcellular shuttle system for the delivery of payloads across the blood-brain barrier (BBB) endothelial cells (45). In other words, clinically approved TfR-BiTE could target and destroy the BBB-endothelial cells and cause significant neurotoxicity. Hence, it is worthy of further research for loco-regional delivery of TfR-BiTE in order to increase its efficacy and decrease the neurotoxicity.

Our results show that TfR-BiTE can mediate the attachment of effector cells with tumor cells to direct polyclonal cytotoxic T cells to kill TfR-positive tumor cells *in vitro* and *in vivo*. TfR-BiTE is a promising tumor-targeting antibody for the treatment of TfR positive solid tumors.

## Data Availability

No datasets were generated or analyzed for this study.

## Ethics Statement

The peripheral blood of healthy donors was obtained from the Blood Center of Hubei Province. All *in vivo* experimental procedures were approved by the Ethics Committee of Tongji Medical College of Huazhong University of Science and Technology.

## Author Contributions

MF, QH, YH, and PL designed the study, analyzed the data. PL and MF wrote the manuscript. MF, QH, ZG, XiaorZ, HL, LZ, HT, and XiaoqZ carried out experiments and statistical evaluation. MF and ZG prepared the TfR-BiTE antibodies. HZ carried out cell culture. PL, YH, and GS provided funding.

### Conflict of Interest Statement

The authors declare that the research was conducted in the absence of any commercial or financial relationships that could be construed as a potential conflict of interest.
