# Supplementary material for: Therapeutic Bispecific T-Cell Engager Antibody Targeting the Transferrin Receptor
Source: Front Immunol. 2019 Jun 21;10:1396. doi: 10.3389/fimmu.2019.01396 (PMC6598450; doi:10.3389/fimmu.2019.01396)
Supplement: Supplementary file 1 [file Data_Sheet_1.docx]

**Supplementary Figure 1**


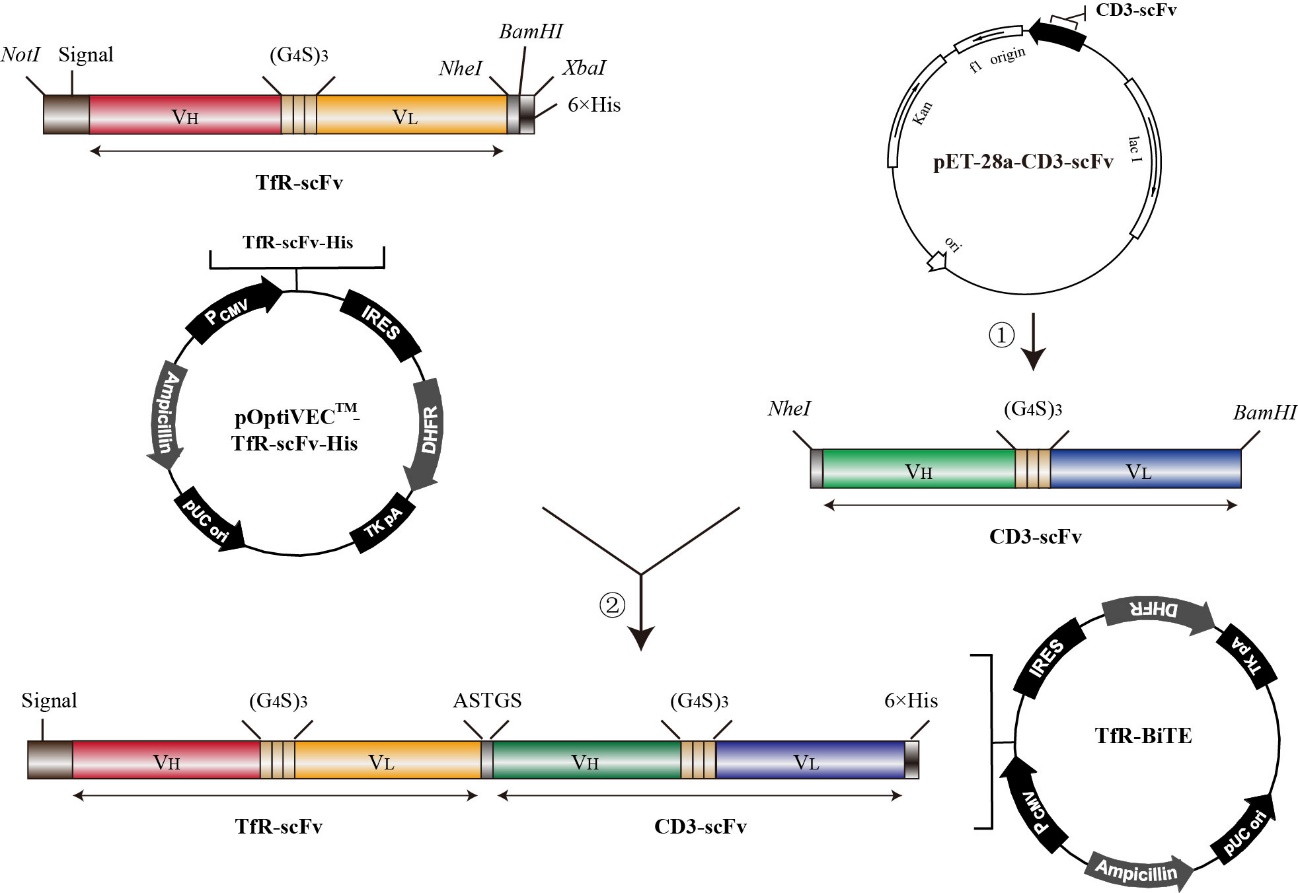


**Supplementary Figure 1.** Schematic diagram of TfR-BiTE construction. ASTGS or (G_4_S)_3_, short linker; *NheI* and *BamHI*, restriction sites. ①②, indicates the process.

**Supplementary Figure 2**


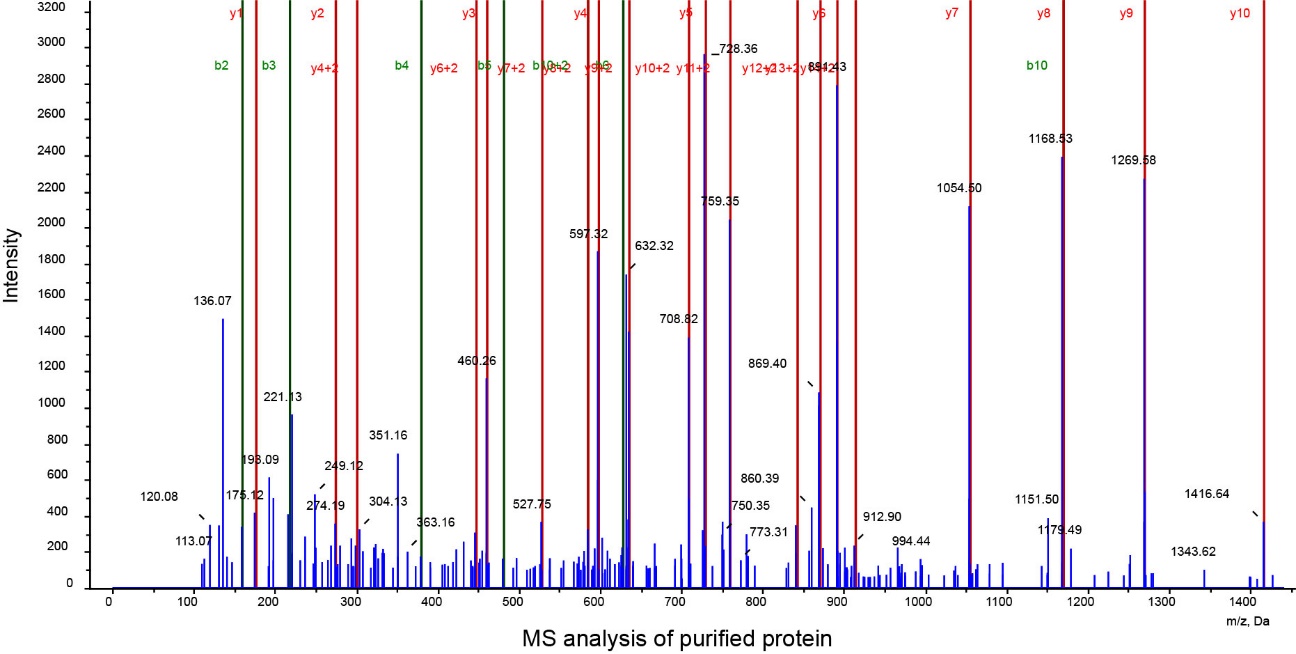


**Supplementary Figure 2.** Mass Spectrometry (LC-MS/MS) analysis of the unique peptide sequence “ASGYTFTNYYMHWVR” of purified protein.

**Supplementary Figure 3**


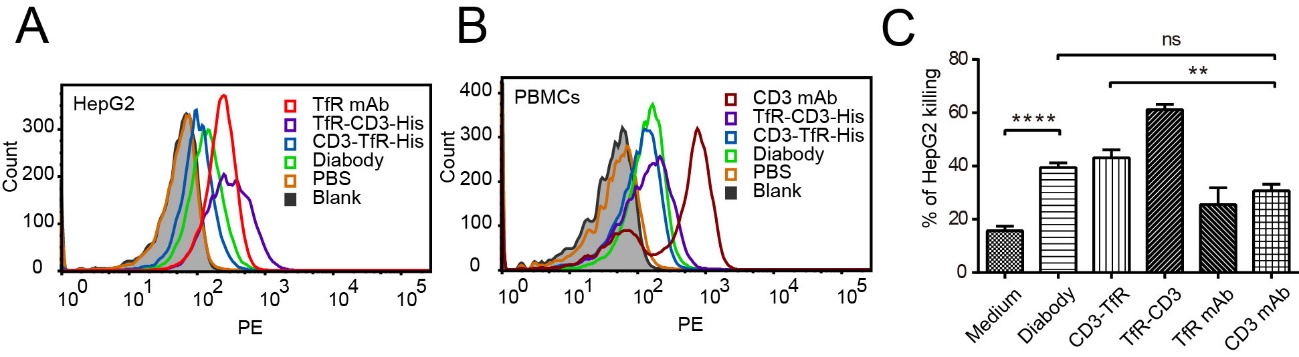


**Supplementary Figure 3:** (A, B) Binding of bispecific antibodies with HepG2 cells (TfR^+^) and PBMCs (CD3^+^) were detected using anti-His tag mAb by flow cytometry analysis. PBS and blank controls were set as the negative controls. (C) CFSE-stained PBMCs and HepG2 cells (E:T=10:1) were incubated with antibodies indicated for 24 h. Then cells were stained with 7-AAD. CFSE^-^ 7-AAD^+^ cells were calculated as the lysed tumor cells.
